# Working from home during the COVID-19 outbreak in Sweden: effects on 24-h time-use in office workers

**DOI:** 10.1186/s12889-021-10582-6

**Published:** 2021-03-17

**Authors:** David M. Hallman, Leticia Bergamin Januario, Svend Erik Mathiassen, Marina Heiden, Sven Svensson, Gunnar Bergström

**Affiliations:** 1 Centre for Musculoskeletal Research, Department of Occupational Health Sciences and Psychology, University of Gävle, Gävle, Sweden; 2 Division of Intervention and Implementation Research in Worker Health, Institute of Environmental Medicine, Karolinska Institutet, SE-171 77 Stockholm, Sweden

**Keywords:** Corona, Telework, Physical activity, Sedentary, Sleep

## Abstract

**Background:**

The COVID-19 pandemic has triggered national recommendations encouraging people to work from home (WFH), but the possible impact of WFH on physical behaviors is unknown. This study aimed to determine the extent to which the 24-h allocation of time to different physical behaviors changes between days working at the office (WAO) and days WFH in office workers during the pandemic.

**Methods:**

Data were collected on 27 office workers with full-time employment at a Swedish municipal division during the COVID-19 outbreak in May–July 2020. A thigh-worn accelerometer (Axivity) was used to assess physical behavior (sedentary, stand, move) during seven consecutive days. A diary was used to identify periods of work, leisure and sleep. 24-h compositions of sedentary, standing and moving behaviors during work and non-work time were examined using Compositional data analysis (CoDA), and differences between days WAO and days WFH were determined using repeated measures ANOVA.

**Results:**

Days WFH were associated with more time spent sleeping relative to awake, and the effect size was large (F = 7.4; *p* = 0.01; η_p_^2^ = 0.22). The increase (34 min) in sleep time during WFH occurred at the expense of a reduction in work and leisure time by 26 min and 7 min, respectively. Sedentary, standing and moving behaviors did not change markedly during days WFH compared to days WAO.

**Conclusion:**

Days working from home during the COVID-19 pandemic in Sweden were associated with longer duration of sleep than days working at the office. This behavioral change may be beneficial to health.

**Supplementary Information:**

The online version contains supplementary material available at 10.1186/s12889-021-10582-6.

## Background

Since the COVID-19 outbreak, working from home (WFH) has become the new normal for many workers with office-based jobs [1]. Recommending and implementing WFH is considered an important action to reduce exposure to virus and thereby decrease the risk of infection [2]. However, the increase in WFH during the COVID-19 pandemic may have both positive and negative impacts on working conditions as well as physical and mental health among employees [3].

Physical (in) activity and sleep are important determinants of health [4] and thus an essential issue in public health recommendations [5]. More physical activity is considered beneficial to health while too much sitting is detrimental. Sleep duration shows a U-shaped relationship with health, where both too much [6] and too little [7] sleep is associated with poor health outcomes, including cardiovascular diseases and mortality. Data collected before and during COVID-19 at the population level suggest that physical behaviors, i.e. different types of physical (in) activity, and sleep, have changed; physical activity has decreased, while sitting and total sleep time have increased [8–10]. One explanation may be that WFH is associated with other physical behaviors than working at the workplace. Thus, although research is sparse, some studies suggest that WFH is associated with changes in the time spent in different physical behaviors [11], but little is known specifically regarding the extent to which physical behaviors of office workers differ between WFH days and days at the office. Also, previous studies may not apply to WFH during the COVID-19 outbreak. First, the extent of WFH has increased largely during the pandemic [12], now including workers with limited previous experience of WFH. Second, as WFH is strongly recommended during the COVID-19 outbreak, and in some cases even mandatory, it is likely not voluntary to the same extent as before the pandemic [13, 14]. Voluntary WFH is often adopted by employees as a strategy to adapt work to private life, motivated by family and household needs [14], and may be practiced mainly by employees having favorable conditions for WFH in terms of family life, household chores and workstation at home. Less voluntary WFH during the pandemic, likely performed even under unfavorable conditions, may be associated with changed physical behaviors because of these contextual differences [15, 16]. More research on the trade-off between positive and negative aspects of WFH is therefore needed as a basis for policy recommendations during and beyond the pandemic.

A day is comprised of 24 h during which time is allocated to different physical behaviors distributed among different domains (e.g. work and leisure). More time can be spent in one behavior only at the cost of reducing time in another behavior. For instance, increasing time in sleep will inevitably lead to less time awake; more time at work will lead to less leisure time; and more time spent being physically active will result in less inactive time. This inherent correlation in time-use data implies that standard statistical procedures cannot be used unless data are first processed using specifically adapted procedures. In Compositional data analysis (CoDA), data are expressed in terms of log-transformed ratios expressing relations between compositional parts (in the present case, different physical behaviors, and sleep), and those ratios can be analyzed using standard statistics [17, 18]. Thus, we used a CoDA approach as a basis for examining how WFH influences the 24-h time-use composition of sitting, standing, moving and sleeping; compared to days working at the office (WAO).

We used wearable technical measurement devices (accelerometers) to accurately determine physical behavior, complemented with a diary identifying work, leisure and sleep time. We used a within-subject repeated-measures design to compare WFH days and WAO days, as this design increases statistical power and controls for between-worker confounding by, e.g. income and skill, which would likely be higher among office workers with extensive WFH than among office workers more confined to the physical office space [19].

The present study focused on office workers working from home during the COVID-19 outbreak in Sweden. We aimed to determine the extent to which 24-h time use differs between days working at the office (WAO) and days working from home (WFH), in terms of wake time relative to sleep, time spent working relative to leisure, and time spent sitting relative to standing and moving.

## Methods

### Design and study population

This observational study on office workers used a within-subject design. Data were analyzed from the first wave of an ongoing cohort evaluating flexible work conditions in different public and private organizations in Sweden. We invited 484 workers from a municipal division (Sektor Livsmiljö Gävle) in Sweden to participate. The division is commissioned to provide for the living environment in the municipality, such as buildings, infrastructure, recreational areas, sport facilities and cultural activities. Data collection was done during May–July 2020. During this period, the Public Health Agency in Sweden issued a number of recommendations to diminish spread of the corona virus, some of which were enforced by the government or local authorities. In general, Sweden had a less restrictive policy than most other European countries, and authorities relied largely on citizens taking a personal responsibility [20]. Initiatives included, e.g. prohibiting more than 50 persons to gather in public places, only serving at the table in restaurants, and distance education for secondary schools and universities. Of particular relevance to the present study, people were strongly recommended to WFH if their work tasks allowed for that. Also, citizens were dissuaded from leaving home if having any symptom indicative of COVID-19, and sick leave from work was allowed without a doctor’s certificate. Since WFH was not strictly mandatory, we had the opportunity to collect data during days WAO as well as days WFH in the same workers.

**Fig. 1:**
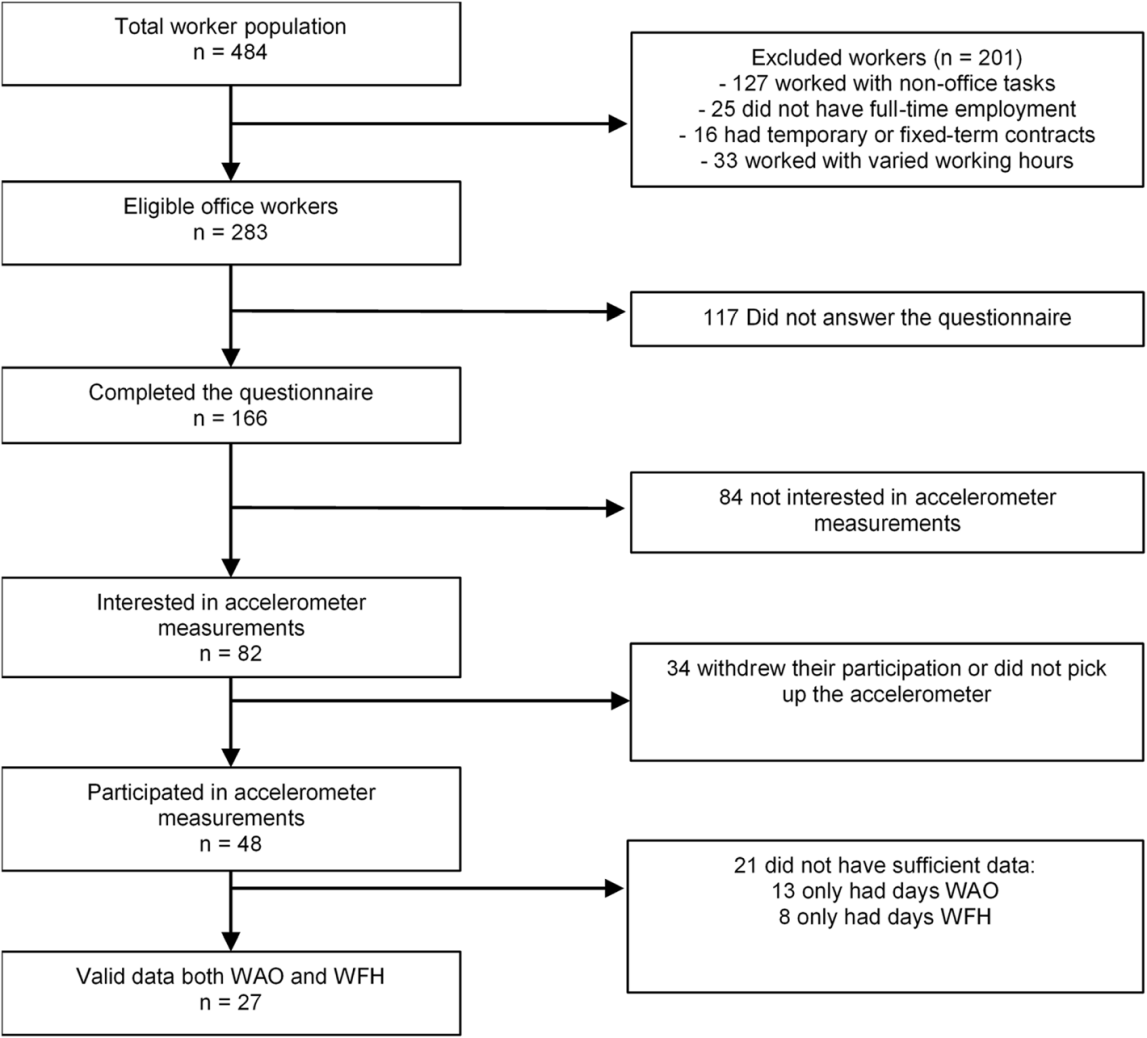
Flow chart of participant recruitment and data collection; WAO: working at the office; WFH: working from home

For this study, we included white-collar workers predominantly involved in office-based tasks, working during business hours, and having a permanent full-time employment contract. They were asked to complete a web-based questionnaire and participate in measurements of physical behaviors (see below). The study was performed in accordance with the Declaration of Helsinki. The Swedish Ethical Review Authority approved the study (decision 2019–06220), and all participants provided their written informed consent. The flow of participants is shown in Fig. 1.

### Measurements

#### Company data

We obtained information from company records regarding age, gender, company position (manager or employee), occupational class (white-collar worker or blue-collar worker), working schedule (business hours or varied working hours [shifting between business hours, evenings and weekends]), work arrangement (flexible time or non-regulated working hours), type of contract (permanent contract/until further notice, temporary employment or fixed-term contracts), and % full-time employment. This information gave the basis for identifying eligible workers.

#### Questionnaires

All workers meeting the inclusion criteria above received a personal link by email, connecting to a web-based questionnaire to be answered within a month. In the following three weeks, the workers received emails with weekly reminders. We asked questions about demographics and personal information including country of birth, education and smoking habits. Work ability was assessed using a valid single item from the work ability index [21], using a response scale from 0 (unable to work) to 10 (work ability at its best). Quantitative demands were assessed using the third version of the Copenhagen Psychosocial Questionnaire – COPSOQ [22]. An index was obtained by averaging three items (middle version, QD1-QD3), with higher values on the 0–100 scale indicating higher demands. Perceived physical and mental exertion were assessed using a numerical rating scale adapted from Borg [23], ranging from 0 (not at all) to 10 (extremely exerted). Wellbeing was assessed using the World Health Organization wellbeing index, WHO-5 [24], ranging from 0 to 100, with higher values representing better wellbeing. Finally, general health was assessed by a valid single item from the Short Form Health Survey, SF36, ranked into 5 categories from excellent to poor [25, 26].

Workers participating in measurements of physical behavior also answered questions addressing to what extent their working situation had changed during the ongoing pandemic. Thus, they rated to what extent they used to work from home prior to the pandemic and to what extent they were working from home right now (i.e. during the pandemic). These questions were answered on a four-point scale, ranging from 1 (to a very high extent) to 4 (not at all). They also answered eight questions inspired by COPSOQ-III [22] regarding perceived changes during the pandemic, compared to before, in workload, work tasks, wellbeing, work performance, influence and support from managers, boundaries between work and private life, and expectations about being available for work-related issues during leisure time. These questions had five response alternatives, i.e. ‘much improved’, ‘somewhat improved’, ‘no change’, ‘somewhat worse’, and ‘much worse’. The questions addressing changes during the COVID-19 pandemic are shown in Additional file 1.

#### Assessment of time use

We assessed physical behaviors over 7 days using one wearable triaxial accelerometer (Axivity AX3, Axivity Ltd., Newcastle, UK) placed on the front of the right thigh, midway between the hip and the knee joint. During a 30-min session, a member of the research team fixed the accelerometer to the skin using double-sided adhesive tape (3 M, Hair-Set, Saint Paul, Minnesota, USA) and Fixomull (Fixomull BSN medical GmbH, Hamburg, Germany), and measured the body weight and height of the participant. Accelerometer data were processed with the Acti4 algorithm using a custom-made MATLAB software. The Acti4 algorithm, identifies different types of physical behaviors with a confirmed good validity [27, 28]. During the full 7-day period, participants were asked to fill in a diary defining their time in bed, as well as working hours and leisure. They were also asked to report if work was done at the office or at home. Workers were only included in further analyses if they reported both days WFH and days WAO during the measurement period. Only days with complete 24 h measurements were included in further analyses.

For the present study, we determined time spent in bed (i.e. sleeping), working, and in leisure based on the diary; and time spent sitting/lying (SED), standing, and moving (i.e. walking, walking stairs, running and cycling) from the accelerometer recordings. Combining these two data sources allowed us to calculate time spent SED, standing and moving separately for work and leisure, for individual WAO and WFH days. We then calculated the mean time in each behavior for WAO and WFH days, to arrive at a composition of WAO and WFH behaviors for each participant. This composition contained seven parts: SED, standing, moving during work (i.e. workSED, workSTAND, workMOVE), the same three behaviors during leisure (i.e. leisSED, leisSTAND, leisMOVE), and sleep.

### Data analysis

#### Time use compositions (CoDA)

We analyzed the 24-h time-use compositions during WAO and WFH using a CoDA procedure, in which the absolute values of the seven compositional parts were transformed into a set of six isometric log-ratios (ILRs). We constructed our set of ILRs to specifically reflect contrasts in behavior that we wished to address, as described further below [29]:

$$ ILR1=\sqrt{\frac{6}{7}}\ln \frac{sleep}{\sqrt[6]{workSED\times workSTAND\times workMOVE\times leisSED\times leisSTAND\times leisMOVE}} $$

$$ ILR2=\sqrt{\frac{9}{6}}\ln \frac{\sqrt[3]{workSED\times workSTAND\times workMOVE}}{\sqrt[3]{leisSED\times leisSTAND\times leisMOVE}} $$

$$ ILR3=\sqrt{\frac{2}{3}}\ln \frac{workSED}{\sqrt[2]{workSTAND\times workMOVE}} $$

$$ ILR4=\sqrt{\frac{1}{2}}\ln \frac{workSTAND}{workMOVE} $$

$$ ILR5=\sqrt{\frac{2}{3}}\ln \frac{leisSED}{\sqrt[2]{leisSTAND\times leisMOVE}} $$

$$ ILR6=\sqrt{\frac{1}{2}}\ln \frac{leisSTAND}{leisMOVE} $$

ILR_1_ represents the ratio of sleeping time to time spent in all other behaviors (i.e. time awake), expressed as a geometric average. ILR_2_ represents, within time awake, the ratio of time working to time spent in leisure. ILR_3_ and ILR_4_ represent time at work spent in sedentary relative to non-sedentary behaviors (ILR3), and – within non-sedentary behaviors – time spent standing relative to time spent moving (ILR4). ILR_5_ and ILR_6_ represent the same two behavior contrasts as ILR3 and ILR4, but for leisure time.

### Statistical analysis

All statistical analyses were performed in the Statistical Package for the Social Sciences (SPSS, version 27.0, IBM, Armonk, NY, USA). Characteristics of the study sample, as well as changes in the extent of WFH and self-reported work characteristics during the pandemic, were described using means and standard deviation (SD) for continuous variables, and frequencies and percentages for categorical data. To compare the 24-h time-use composition between WAO and WFH days, we performed a repeated-measures multivariate analysis of variance (MANOVA), using type of day (two levels, WAO vs. WFH) as a within subject factor and all six ILRs as dependent variables. We also calculated mean differences with 95% confidence intervals for each of the six ILRs separately. In all analyses, partial eta square (η_p_^2^) was used as a measure of effect size, with benchmark values of 0.099, 0.0588, and 0.1379 indicating small, medium, and large effect sizes, respectively [30] .

## Results

### Flow of participants

In the total population of 484 workers, 283 met the inclusion criteria, and 166 of them completed the questionnaire (response rate 59%). Of the 166 office workers, 82 were interested in the accelerometer measurements, 48 took part in the accelerometer measurements, and 27 of them provided valid records including both days WAO and days WFH. The flow of participants is shown in Fig. 1.

### Characteristics of the study population

The descriptive statistics of the participants with accelerometry (final sample), the respondents in the questionnaire, and the target population of all office workers in the organization who met the inclusion criteria are shown in Table 1. All workers were classified as white-collar, had a permanent position, and worked full time (about 40 h per week) according to company records. The final study sample contained a higher proportion of females compared to the respondents to the questionnaire and the target population. Also, the final study sample only contained workers born in Sweden, while the respondents to the questionnaire included 8% born outside Sweden. No marked differences were observed in age, position, smoking, work ability, work demands (quantitative demands and exertion), wellbeing, and general health. All office-workers had private offices.

**Table 1:** Characteristics of the target population, respondents to the questionnaire, and participants with accelerometry in the study

	Participants with accelerometry (*n* = 27)		Respondents, questionnaire (*n* = 166)		Target population (*n* = 283)	
	N (%)	Mean (SD)	N (%)	Mean (SD)	N (%)	Mean (SD)
Sex (female)^a^	22 (81.5)		106 (63.9)		182 (64.3)	
Age (years)^a^		43.4 (9.9)		44.5 (11.3)		44.5 (11.4)
Country of birth^b^						
Sweden	27 (0.0)		150 (90.4)			
Other	0 (0.0)		14 (8.4)			
Education^b^						
Secondary education	2 (7.4)		18 (10.8)			
Higher education	25 (92.6)		147 (88.5)			
Manager position^a^	2 (7.4)		17 (10.2)		24 (8.5)	
Work arrangement						
Flextime	25 (92.6)		150 (90.4)		259 (91.5)	
Non-regulated time	2 (7.4)		16 (9.6)		24 (8.5)	
Smokers^b^	0 (0.0)		6 (3.6)			
Work ability (0–10)^b^		8.4 (1.7)		8.2 (1.5)		
Quantitative demands (0–100)^b^		53.4 (19.6)		49.6 (21.0)		
Physical exertion (0–10)^b^		1.2 (1.93)		1.2 (1.7)		
Mental exertion (0–10)^b^		6.8 (1.5)		6.7 (1.7)		
Well-being (0–100)^b^		52.9 (15.2)		55.1 (18.7)		
General health^b^						
excellent	0 (0.0)		7 (4.2)			
very good	10 (37.0)		51 (30.7)			
good	12 (44.4)		72 (43.4)			
fair	4 (14.8)		33 (19.9)			
poor	1 (3.7)		2 (1.2)			
Body mass index^c^		25.3 (4.8)				

*N* number of workers, *SD* standard deviation

^a^Assessed through company data

^b^Self-reported information from online questionnaire

^c^Objectively measured

### Self-reported changes in working conditions during COVID-19

**Fig. 2:**
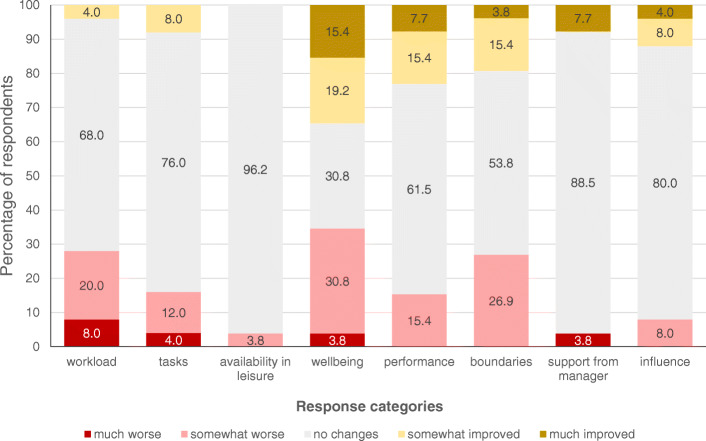
Self-reported changes (percentage of respondents) in workload, work tasks, expectations on availability during leisure, wellbeing, work performance, boundaries between work and private life, support from managers, and influence at work during the COVID-19 pandemic. N = 27

The self-reported extent of WFH increased during the COVID-19 outbreak compared with before the pandemic. Before the pandemic, only 12% of the workers reported WFH to a high or very high extent, while this proportion increased to 76% during the COVID-19 outbreak. Self-reported changes in working conditions during the pandemic are shown in Fig. 2. For all investigated items, most workers reported no change during the pandemic; about one third of the workers reported that workload, wellbeing, and boundaries between work and life have changed for the worse; and more than one in five workers reported improvements in wellbeing, performance and work-life boundaries.

### Time use during days working at the office and days working from home

**Fig. 3:**
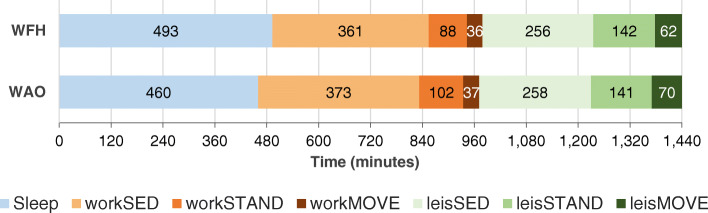
Mean time (minutes/day) during days working from home (WFH) and days working at the office (WAO) spent in sleep (blue), and in sedentary, standing and moving during work (orange) and leisure (green)

On average, valid data on physical behavior were obtained for 3.8 workdays per worker (2.1 days WAO and 1.7 days WFH). The mean (SD) of times spent in sleep, work, leisure, and physical behaviors within work and leisure are shown in Table 2 and Fig. 3. On average, the workers spent 34 min more in sleep, 26 min less working, and 7 min less in leisure during days WFH than during days WAO. Time spent in physical behaviors only differed to a minor extent between WFH and WAO; most notably with days WFH showing 12 min less sedentary time at work, and 7 min less time moving during leisure than days WAO. About 80% of the time moving was spent walking, both while working and during leisure, and both on days WAO and WFH (not in table).

**Table 2:** Arithmetic means with standard deviation between workers (SD) of time (minutes/day) spent in the seven investigated physical behaviors during all observed days, days working at the office (WAO days) and days working from home (WFH days)

	All days	WAO days	WFH days
	Mean (SD)	Mean (SD)	Mean (SD)
Sleep	477 (57)	460 (50)	494 (63)
Work	499 (185)	512 (165)	486 (205)
Sedentary	367 (101)	373 (86)	361 (116)
Standing	95 (63)	102 (63)	88 (63)
Moving	37 (22)	37 (17)	36 (27)
Leisure	465 (144)	468 (128)	461 (159)
Sedentary	257 (61)	258 (50)	256 (71)
Standing	142 (51)	141 (44)	143 (58)
Moving	66 (32)	70 (34)	62 (30)

The repeated-measures MANOVA of relative time use (i.e. of the six ILRs together) indicated that days WFH and days WAO differed in their overall 24-h time-use composition (F = 2.45; *p* = 0.06; η_p_^2^ = 0.41). Univariate models (Table 3) indicated a large effect of WFH on sleep time relative to time awake (ILR_1_); time spent sleeping relative to awake increased during days WFH compared with days WAO (Table 2).

**Table 3:** Statistical results from the univariate models on time use expressed as isometric log ratios (ILR). N = 27

Effect of day type	F-value	*P*-value	η_p_^2^
ILR_1_: sleep/wake	7.40	0.01	0.22
ILR_2_: work/leisure	0.29	0.59	0.01
Behaviors at work			
ILR_3_: sedentary/non-sedentary	0.79	0.38	0.03
ILR_4_: standing/moving	0.02	0.89	0.00
Behaviors at leisure			
ILR_5_: sedentary/non-sedentary	0.48	0.49	0.02
ILR_6_: standing/moving	1.14	0.30	0.04

η_p_^2^: partial eta squared

**Fig. 4:**
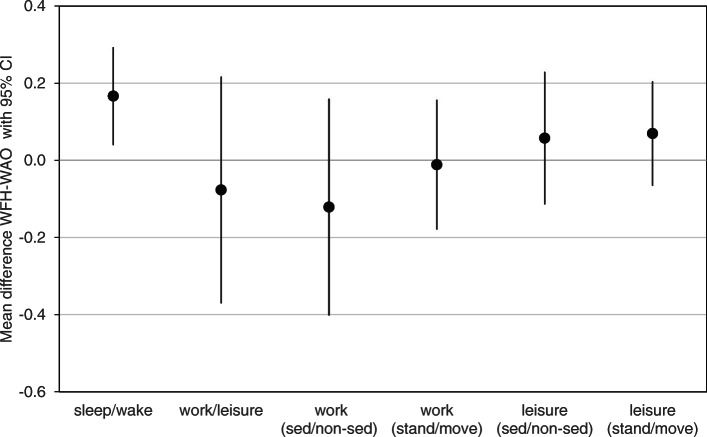
Mean difference between working from home (WFH) and working at the office (WAO) with 95% confidence interval (CI) for each of the six ILRs (x-axis)

Figure 4 shows the mean difference in the 24-h composition of behaviors between days WFH and WAO, expressed in terms of the six ILRs described in section 2.3.1. The estimates indicate, as confirmed in the univariate analysis above (Table 3), that WFH is clearly associated with more time spent sleeping relative to awake. Although confidence intervals include 0 for all other ILRs (i.e. whether WAO and WFH do, indeed, differ is uncertain), it is of note that WFH showed a trend towards less time spent working relative to leisure.

## Discussion

We conducted a within-subject repeated-measures study to determine the extent to which working from home (WFH) during the COVID-19 outbreak influences the 24-h time-use composition in office workers, compared to days when workers go to the office (WAO). Using a CoDA approach for processing and analyzing behavior compositions, we found that days WFH were associated with more time spent sleeping relative to awake, while behaviors during work and leisure did not change markedly, compared with days WAO.

The observed 34 min increase in sleep time during days WFH occurred at the expense of reduced work (26 min) as well as leisure time (7 min). The CoDA showed that the relative proportions of work and leisure “within” the time awake were almost similar for days WAO and WFH (Table 3). This corroborates previous cross-sectional surveys conducted during the pandemic, suggesting that WFH during the pandemic is associated with longer sleep time [31] and shorter working hours [32] than before the restrictions. A longer sleep time during confinement was also reported in a survey of 14,000 workers from 11 countries [33]. A possible explanation for some waking hours (in our study 34 min) being reallocated to sleep during days WFH is that workers take the opportunity to sleep longer when they do not need to commute to the workplace in the morning. Moreover, the trend towards reduced working time from 512 min/day (8.5 h) on WAO days to 486 min (8.1 h) during days WFH suggests that people tend to work less overtime during WFH days, although this needs to be confirmed in larger studies. This trend is in contrast with previous pre-COVID-19 studies, reporting WFH to be associated with longer working hours [34].

We could not confirm that WFH had any marked influence on proportions of time awake spent in sedentary behavior, standing and moving in our sample of office workers. This is in contrast to a recent survey during the pandemic, reporting that respondents who were now working from home had more self-reported sedentariness than respondents whose working conditions were unchanged [35]. However, among our participants, behaviors at work might have changed during the pandemic. We did not have any pre-COVID-19 data in this population to further examine that issue. Population-based studies indicate that the pandemic may have resulted in reduced physical activity [9, 36], although representative national data confirming that are currently lacking.

Recent 24-h movement guidelines [5] recommend that in order to reduce health risks, adults should spend 7–9 h every night in sleep, at the most 8 h per day sedentary, and at least 150 min per week in moderate to vigorous physical activity, along with several hours of light activity and standing. On average, our sample was a bit closer to achieving a balanced 24-h time use according to these guidelines during days WFH than on days WAO; in particular, sleep increased relative to wake time, which may have beneficial implications for health and wellbeing [7].

However, we also found that our sample of office workers spent more than 10 h per day being sedentary – in conflict with the cited guidelines – and that 59% of this time was accumulated during work. A total sedentary time of this size, which is consistent with other studies of office workers [37, 38], has been associated with considerable health risks and early mortality, particularly among those not also engaged in physical activity of sufficient intensities [4, 39]. Thus, our results suggest that policies should encourage office workers to be more active, regardless of whether they work from home or go to the office.

Although the average worker did not report any major changes in working conditions, performance, wellbeing and boundaries between work and private life during the pandemic compared to before (see Fig. 2), we found a considerable heterogeneity between workers in these outcomes; some workers perceived changes to be beneficial while others reported negative changes. For instance, 28% reported that workload had changed for the worse. Although the factors underlying these results remain unknown, our findings suggest that some workers could be in need of additional support from the organization in order to cope with their work situation during the pandemic [40], and that it would be important for the organization to be able to identify and help these workers. When the COVID-19 pandemic is eventually over, it will be important to compare 24-h WFH behaviors with evidence from before the pandemic, so as to understand the extent to which COVID-19 experiences have led to a “new normal”. Also, more research is needed on the trade-off between negative and positive effects of WFH, together with other factors of relevance to work environment and health, to support recommendations for WFH after the pandemic.

The present study has several strengths, including the access to accurate exposure data based on accelerometry, and the use of a within-subject design which minimized risk of bias and confounding. The quite small sample size is a limitation, since it precluded us from detecting small, yet relevant, effect sizes with a sufficient certainty, and limited the opportunity to investigate possible effect modifiers, such as employment, age, gender, and occupational status. We only collected data in one organization, which limits the generalizability of the results. Also, we only collected data during an early phase of the pandemic (May–July 2020), and behaviors may have changed during later phases. Nevertheless, our results may extend to WFH practices after the pandemic better than results of studies addressing workers strictly forced to work from home, since our data were collected in a population with high occurrence of WFH, yet with an option of also working at the office.

## Conclusion

We found that office workers during the COVID-19 outbreak in Sweden spent more time sleeping relative to awake during days when they worked from home, compared to days when they went to the office, while physical behaviors during work and leisure did not change markedly. The observed changes in 24-h time use during days working from home may be beneficial to health. Some workers in this sample reported that their workload, performance and wellbeing changed for the worse during the pandemic, suggesting that they would need interventions to prevent potential health risks.

## Supplementary Information

**Additional file 1.**

## Data Availability

The datasets used and/or analyzed during the current study are available from the corresponding author on reasonable request.
