# Supplementary material for: Working from home during the COVID-19 outbreak in Sweden: effects on 24-h time-use in office workers
Source: BMC Public Health. 2021 Mar 17;21:528. doi: 10.1186/s12889-021-10582-6 (PMC7968563; doi:10.1186/s12889-021-10582-6)
Supplement: Supplementary file 1 — Additional file 1. [file 12889_2021_10582_MOESM1_ESM.docx]

**Additional file 1.**

**The following questions concern your working situation during the ongoing COVID-19 pandemic.**

|  | To a very high degree | To a somewhat high degree | To some degree | Not at all |
| --- | --- | --- | --- | --- |
| Prior to the Covid-19 pandemic, to what extent did you work remotely? |  |  |  |  |
| To what degree have you taken advantage of your opportunity to work remotely during the ongoing Covid-19 pandemic? |  |  |  |  |

**The following questions concern whether your work and wellbeing have changed due to the Covid-19 pandemic.**

| Due to the Covid-19 pandemic… | Yes, much improved now | Yes, somewhat improved now | No, no particular changes | Yes, somewhat worse now | Yes, much worse now |
| --- | --- | --- | --- | --- | --- |
| Has your workload changed? |  |  |  |  |  |
| Have your work tasks changed? |  |  |  |  |  |
| Have expectations regarding your availability by phone or your reading of work-related email during leisure time changed? |  |  |  |  |  |
| Has your wellbeing at work changed? |  |  |  |  |  |
| Has your work performance changed? |  |  |  |  |  |
| Have the boundaries between your work and private life changed? |  |  |  |  |  |
| Has the support and help you receive from your immediate supervisor changed? |  |  |  |  |  |
| Has your ability to influence important decisions regarding your work changed? |  |  |  |  |  |
